# Leaching of Potentially Toxic Elements from Paper and Plastic Cups in Hot Water and Their Health Risk Assessment

**DOI:** 10.3390/toxics13080626

**Published:** 2025-07-26

**Authors:** Mahmoud Mohery, Kholoud Ahmed Hamam, Sheldon Landsberger, Israa J. Hakeem, Mohamed Soliman

**Affiliations:** 1 Department of Physical Science, College of Science, University of Jeddah, Jeddah 80327, Saudi Arabia; kahamam@uj.edu.sa; 2 Nuclear Engineering Teaching Lab Walker Department of Mechanical Engineering, University of Texas at Austin, Austin, TX 78712, USA; s.landsberger@mail.utexas.edu; 3 Department of Biological Sciences, College of Science, University of Jeddah, Jeddah 80327, Saudi Arabia; ijhakeem@uj.edu.sa; 4 Egypt Second Research Reactor, Egyptian Atomic Energy Authority, Cairo 13759, Egypt; soliman.ma@gmail.com

**Keywords:** paper and plastic cups, potentially toxic elements, leaching, health risk assessment, single-use products, ICP-MS

## Abstract

This study aims to investigate the release of potentially toxic elements from disposable paper and plastic cups when exposed to hot water, simulating the scenario of their use in hot beverage consumption, and to assess the associated health risks. By using ICP-MS, twelve potentially toxic elements, namely As, Ba, Cd, Co, Cr, Cu, Mn, Mo, Pb, Sb, V, and Zn, were determined in leachates, revealing significant variability in mass fractions between paper and plastic cups, with plastic cups demonstrating greater leaching potential. Health risk assessments, including hazard quotient (HQ) and excess lifetime cancer risk (ELCR), indicated minimal non-carcinogenic and carcinogenic risks for most elements, except Pb, which posed elevated non-carcinogenic risk, especially in plastic cups. Children showed higher relative exposure levels compared to adults due to their lower body weights (the HQ in children is two times greater than in adults). Overall, the findings of the current study underscore the need for stricter monitoring and regulation of materials used in disposable cups, especially plastic ones, to mitigate potential health risks. Future investigations should assess the leaching behavior of potentially toxic elements under conditions that accurately mimic real-world usage. Such investigations ought to incorporate a systematic evaluation of diverse temperature regimes, varying exposure durations, and different beverage types.

## 1. Introduction

The beginnings of the plastic industry began in 1856 when Parkes created his material called parkesine, which was cellulose that had been treated with nitric acid to create pyroxillin which was then dissolved in alcohol, which was then followed by the first Baekeland in 1907, who used a combination of natural cellulose and synthetic materials to produce bakelite [1]. In the early 1950s, plastic was produced at only a few million tons per year, while by 2025 it has increased to approximately 500 million tons [2]. Plastic pollution in the marine environments and landfills has been described as a global threat that includes “changes to carbon and nutrient cycles; habitat changes within soils, sediments, and aquatic ecosystems; co-occurring biological impacts on endangered or keystone species; ecotoxicity; and related societal impacts” [3]. A review of the characteristics of plastic pollution in the environment has shown how exacerbated ecosystems have become in the last few decades [4]. Recently, the occurrences, sources, fate, and impacts of plastic materials on aquatic ecosystems and human health in global perspectives have been delineated [5], while the description of the metamorphosis of megaplastics to nanoplastics has been deliberated [6].

Plastics have a wide range of applications across various industries, including their significant role in food packaging. Disposable cups, in particular, represent one of the most widely utilized plastic products in food packaging processes [7,8]. Even disposable paper cups incorporate plastic materials in their structure. Typically, plastic (often polyethylene) is used as inner linings for paper cups to add resilience and prevent leakages of beverages, especially hot liquids [7]. Typically, paper cups are manufactured as a paperboard substrate with a weight of 150 to 350 g/m^2^ paired with a polyethylene liner of 8 to 20 g/m^2^, with a thickness of approximately 50 μm [9]. Despite their convenience, disposable paper and plastic cups are rarely recycled and are ultimately disposed of in landfills [10,11]. As a result, their widespread use contributes to environmental pollution. Estimates suggest that the annual single-use consumption of disposable cups will approach 300 billion by 2025 [9]. In the Kingdom of Saudi Arabia, the paper cups market is experiencing substantial growth, driven by the expanding food and beverage service sector [12]. This growth is fueled by progressively more permissive social norms and the robust demand for ready-to-drink beverages among Hajj and Umrah pilgrims and airline services, as well as addressing the needs of social gatherings and recreational activities.

The intersection of single use of disposable cups and plastic pollution has the potential to have an everlasting impact on the various ecosystems and eventually on human health. In our previous study, 36 chemical elements were identified in paper and plastic cups, including naturally occurring radioelements Th and U and potentially toxic elements such as As, Ba, Cr, Cu, Mn, and V [8]. These findings highlighted their potential risks to the environment and ecosystem and emphasized the importance of further investigation into the sources, leaching behavior, and impacts of these elements to mitigate their environmental and ecological effects. Potentially toxic elements, including Cr, Cd, Ni, and Pb, were detected in the polyethylene lining of paper cups [13].

Several studies have confirmed the potential leaching of hazardous substances such as anions (e.g., Cl^−^, F^−^, and SO_4_^2−^), organic compounds (e.g., phthalates), potentially toxic elements (e.g., Cr, Cd, Pb, Ni, and As), and microplastics from disposable paper and plastic packing [11,13,14,15]. These findings highlight the significant risks these products pose to human health. Research work conducted by Khaled et al. has investigated the impact of contact duration on the leaching of heavy metals from plastic cups into various foodstuffs [16]. In the last several years, growing concern has emerged regarding the leaching of chemical additives and hazardous substances from paper and plastic food packaging. Despite this increasing attention, there are limited literature data available on the leaching of potentially toxic elements from paper cups [11,17]. Publications on this subject are both rare and inconsistent, showing significant variation in the reported results. This highlights a pressing need for systematic studies and investigations to better understand the risks posed by these packaging materials and to provide reliable data that can guide safer practices and regulations.

As part of an international effort, the University of Jeddah has launched an extensive project to investigate the ecological and public health consequences of single-use paper cups. This initiative aims to support decision-makers in managing their use and monitoring their health and environmental effects. In alignment with our previous work concerning the environmental impact of different single-use paper and plastic cups [8], as well as other disposable plastic products [18,19], the objective of the current work is to examine the leaching of selected potentially toxic elements from paper and plastic cups under the conditions simulating the serving of hot beverages using hot water. Furthermore, this study aims to evaluate the health impact resulting from exposure to these potentially toxic elements present in the leachates of paper and plastic cups.

## 2. Materials and Methods

### 2.1. Sampling

In total, 20 pristine commercially available paper cups in various colors, produced by different manufacturers, were sampled from local markets in Saudi Arabia. Additionally, five plastic cups were also sampled. The large sample size of paper cups encompasses the broad market diversity of manufacturers and styles. Since plastic cups on the local market—identified on-cup by the manufacturer as polypropylene (PP)—are far fewer and more uniform, we limited that set to five samples. No special treatments or preparation procedures were applied to the collected samples. They were merely washed three times with ultrapure water to remove any dust or loose contaminants from their surfaces. A sample photo of the investigated cups is shown in Figure S1 of the Supplementary Materials.

### 2.2. Fourier Transform Infrared Spectrometer (FT-IR) Analysis

FT-IR (Nicolet is10, Waltham, MA, USA) was employed to identify the type of polymeric thin film laminated in paper cups. Prior to the FT-IR analysis, the plastic liner was separated from paper cups by using the procedures outlined by Ranjan et al. [1]. Briefly, five randomly selected paper cups were immersed in warm water (30–40 °C) until the cups were completely wet. Random subsampling was performed with MS Excel by using the “index” and “randbetween” functions. The isolated hydrophobic thin films (Figure S2) were carefully separated and gently washed several times with warm distilled water to remove any residual paper. Then, FT-IR analysis was then performed in the spectral range of ~4000–400 cm^−1^. The obtained spectra were compared with the literature data to identify the type of plastic.

### 2.3. Leaching Experiment

All leaching experiments assessing the release of potentially toxic elements from paper and plastic cups into hot water were performed following previously established procedures [11,13,17]. A double-distilled water unit (Borosil Glass Double Distillation Unit, India) was used to prepare fresh and pyrogen- and organic impurity-free ultrapure water (conductivity < 1 µs/cm). A sample of this water was boiled (~100 °C) in a glass conical flask, and then 200 mL were poured into each cup (initial temperature ~95–100 °C). The water was left for 15 min to simulate the scenario of serving hot drinks in paper and plastic cups. Throughout the 15-minute leaching period, cups were left uncovered to mimic typical consumer use. The period of 15 min was chosen based on a previous report indicating that most individuals prefer to consume their beverages within this time frame, and the temperature of hot drinks reaches equilibrium with the ambient level within that period [1]. Cold water, which had been boiled beforehand, was poured into paper and plastic cups to serve as blank samples for comparison with the effect of hot water samples on the corresponding cups. All experiments were carried out in triplicate. Immediately after collection, leachate samples were transferred into acid-cleaned glass bottles. To preserve the trace elemental composition and prevent any adsorption of metals on bottle walls, the samples were acidified by using high-purity nitric acid. Specifically, we used Suprapur^®^ nitric acid (65%, Merck, Darmstadt, Germany), which is specifically processed to minimize metal impurities. Before acidification, the concentrated acid was diluted with ultrapure water to prepare a 1% (*v*/*v*) working solution. For each 100 mL of leachate, 1.0 mL of this 1% nitric acid solution was added, resulting in a final acid concentration that is sufficient to maintain the metals in solution without introducing significant contamination.

### 2.4. Elemental Analysis of the Leachates

Elemental analysis of the leachate samples was conducted by using Triple Quadrupole Inductively Coupled Plasma Mass Spectrometry (TQ ICP-MS), Thermo Scientific, Inc., Waltham, MA, USA. The operating parameters of the instrument were optimized for the best peak/noise ratio. The key parameters can be briefly summarized as the following: coolant gas flow rate: 14 L/min; auxiliary gas flow: 0.8 L/min; RF power: 1550 W; and nebulizer gas flow rate: 1.08 L/min. The utilized standard method for elemental analysis by TQ-ICP-MS is ASTM D5673−16 [20]. The limits of detection for the determined elements are shown in Table S1. As a quality control procedure, standard reference solutions (Sigma-Aldrich, Burlington, MA, USA) containing 0.5 mg/L of each element of interest were treated and diluted to match the mass fraction of the elements in the investigated samples. The relative standard deviation for the standard solution was less than 10% for all the investigated elements.

### 2.5. Risk Assessment

Ingestion is the primary exposure pathway for the leachates of paper and plastic cups, as harmful substances can be released into beverages and subsequently consumed by individuals. According to the Environmental Protection Agency of the USA, the average daily intake (*ADI*) of the investigated elements can be calculated using the following equation [17,21]:

(1) $$ADI=\frac{C\times IR\times EF\times ED}{BW\times AT}$$

where *C* is the mass fraction of the element of interest in the leachate (mg/L); *IR* refers to consumption rate (amount of hot beverages per day, l/day); *EF* represents the exposure frequency (days/year); *ED* is the exposure duration (years); *BW* is the standard body mass (70 kg); and *AT* indicates the average exposure time (days). For carcinogens, AT=70 (human life expectancy)×365, while for non-carciongens, AT=ED×365. The exposure parameters shown in Equation (1) were obtained from the literature [17,22] and presented in Table 1.

Hazard quotient (*HQ*) was employed to assess the potential non-carcinogenic risk due to exposure to potentially toxic elements during a human lifetime. An *HQ* can be estimated by dividing the *ADI* by a specific reference dose (*RfD*) for a specific element according to the following equation [23,24,25]:

(2) $$HQ=\frac{ADI}{RfD}$$

where *RfD* is expressed in mg/kg_bw_/day. The subscript “bw” refers to human body weight.

The excess lifetime cancer risk (*ELCR*) was applied for evaluating the probability of cancer pathogenesis during a human life expectancy of 70 years. It can be expressed using the following formula [23,24,25]:

(3) ELCR=ADI×SFO

where *SFO* represents the slope factor in units of (mg/kg_bw_/day)^−1^, which is a plausible upper-bound estimate of the probability of a response per unit oral intake of a carcinogen over a lifetime [25].

## 3. Results

### 3.1. Identification of Lining Material

**Figure 1:**
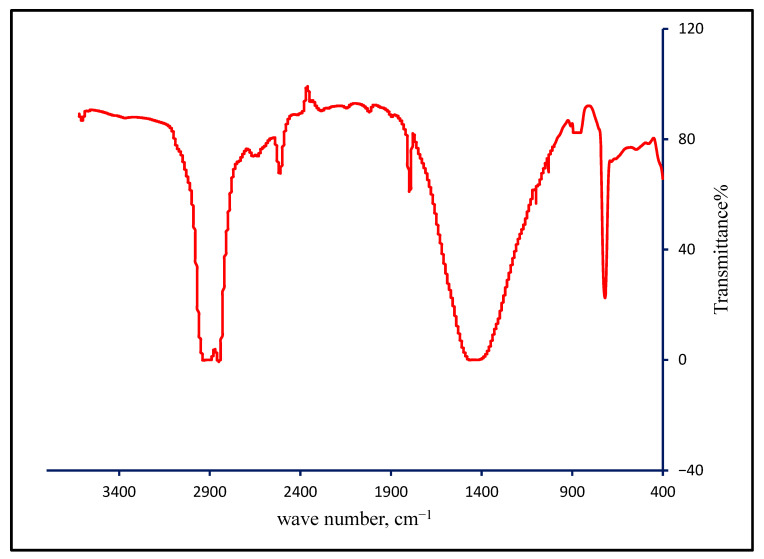
Typical FT-IR spectrum of the inner liner plastic film.

FT-IR examinations were conducted to identify the type of plastics utilized as an inner liner in paper cups. All examined films displayed a typical FT-IR transmission pattern, an example of which is shown in Figure 1.

The FT-IR spectra matched well with bands associated with the methylene group, including asymmetric C–H stretching, symmetric C–H stretching, and methylene rocking at 2932, 2850, and 719 cm^−1^, respectively. The broad band at 1467 cm^−1^ can be attributed to the methylene scissoring bands at 1462 and 1472 cm^−1^. The confirmed presence of the methylene group indicates that the investigated liners are composed of polyethylene [18]. Weak absorption bands at 3604 and 2513 cm^−1^ can be attributed to O–H and S–H stretching, respectively, while the strong band at 1796 cm^−1^ is associated with C–O stretching. Additionally, the weak bands observed at 1030 and 1107 cm^−1^ can be attributed to cellulose attached to the thin polyethylene film separated from paper cups [13].

### 3.2. Elemental Analysis of Leachate in Hot Water

**Figure 2:**
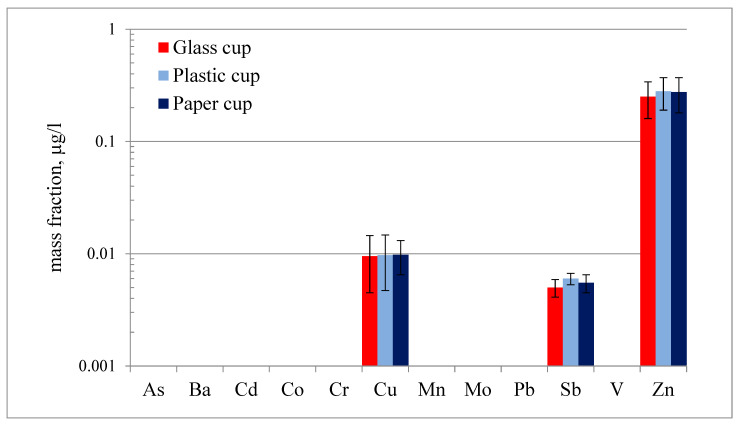
Elemental analysis of control samples. Data are presented as mean ± standard deviation (*n* = 3).

Characterizing the leachates from plastic and paper cups can provide insight into potential health and environmental impacts. ICP-MS was utilized for assessing the mass fraction of 12 potentially toxic elements (As, Ba, Cd, Co, Cr, Cu, Mn, Mo, Pb, Sb, V, and Zn) in the leachate from plastic and paper cups when exposed to hot water. To ensure accurate assessment, the mass fractions of the same elements were determined in three different control (blank) samples. The first control sample is cold water, which had been boiled beforehand and allowed to cool in a glass container, while the second and third ones are the same cooled water poured in plastic and paper cups, respectively. A blank stored in a glass container served as the baseline for the quality of the ultrapure water used in the current study. In contrast, blanks prepared by storing the same water in plastic and paper cups allowed us to assess any leachability of tested elements from these materials under cold water conditions. Analytical determinations were carried out by analyzing at least three samples for each blank type. This approach allowed us to evaluate the potential influence of ambient temperature water on the leaching of tested elements from these materials. This approach helps us isolate the effect of water under ambient conditions on leaching of the elements of interest. The analysis results of blank samples are shown in Figure 2. Among the 12 elements of interest, only three elements were detected in the control samples—Cu, Sb, and Zn. The mass fractions of these elements are very low, ranging from 0.005 (for Sb) to 0.280 (for Zn) µg/L. The statistical analysis utilizing a one-way ANOVA test was conducted to evaluate whether significant differences exist in the mean concentrations of the examined element across three media types: glass, plastic, and paper. The results revealed a *p*-value of 0.99, which exceeds the conventional significance threshold of 0.05. This indicates that the observed variations in elemental concentrations are statistically insignificant and likely due to random variability. This finding implies that exposure to cold water does not significantly influence the extraction of these elements from plastic and paper cups.

The determined elements and their mass fractions in leachates from plastic and paper cups that were in contact with hot water for 15 min are shown in Table 2. These data reveal some noteworthy findings. Overall, the determined elements showed significant variation with respect to mass fractions, spanning from a few ng/L to several tens of µg/L. Notably, Pb and Zn were recorded at comparatively high levels, with median values of 14.4 and 10.8 µg/L for Zn in paper and plastic cups, respectively, and 37.2 µg/L for Pb in plastic cups. Conversely, Sb exhibited the lowest mass fraction (median = 0.009 µg/L) in the leachates of paper cups and was not detected in plastic cup leachates. Similarly, Mo was exclusively detected in the leachates of paper cups, with a median value of 0.055 µg/L. The substantial standard deviations observed across nearly all determined elements in the investigated leachates, even within cups of the same type, highlight significant variability in the mass fractions of these elements. This variability may be attributed to differences in material composition, likely influenced by the quality of raw materials and variations in production processes [18]. This suggests that raw materials and production methods may play a critical role in the extent of elements leaching from paper and plastic cups.

Our previous investigations into the chemical composition of paper and plastic cups have demonstrated that the material of paper cups contains significantly higher concentrations of investigated elements compared to plastic cups [8]. However, contrary to these findings, the leachate from plastic cups exhibits a higher mass fraction of the vast majority of the tested elements, highlighting an increased propensity for leaching from plastic cups when exposed to hot water. Notably, the median concentration of Pb in plastic cups (37.2 µg/L) exceeds that in paper cups (1.38 µg/L) by a factor of more than 26. In general, the abundance of the investigated elements in leachates from paper and plastic cups follows the following ascending orders (based on the median values of mass fractions): Co = Sb < Cd < V < As < Mo < Cu < Cr < Ba < Pb < Mn < Zn (for paper cups), Co < As < Cd < V < Cr < Cu < Mn < Ba < Zn < Pb (for plastic cups).

The presence of such potentially toxic elements in the examined leachates confirms that disposable paper and plastic cups can be considered as an emerging source of chemical pollution.

### 3.3. Comparison with the Literature Data

To contextualize the findings and highlight their significance, this section compares the results of the present study (presented in Table 2) with a few existing studies on the leaching of elements from paper and plastic cups (as summarized in Table 3). The mass fractions of As, Cr, Co, and Cu observed in the present study align with the data reported by Akhdhar et al. [2], who investigated the release of potentially toxic elements from paper cups exposed to hot water. However, the present study reports significantly lower concentrations of V, with values approximately 10 times lower than those documented by Akhdhar et al. (1.06–1.55 µg/L), and it compares well with those reported by Zeng et al. [3]. This study showed that the variation in elemental content does not differ significantly for most of the determined elements, as indicated by the small standard deviations associated with the reported mass fractions. Investigations by Ranjan et. al. [13] demonstrated a significant variation in the elemental composition of laminated polyethylene in paper cups sourced from different manufacturers. For example, the Mn content ranges widely from approximately 4 to 500 µg/L. Such substantial variation is anticipated to impact the Mn concentrations in the leachates. The high concentration of Pb reported in the current study, with median values of 1.38 and 37.2 µg/L for paper and plastic cups, respectively, is comparable to the literature data, which range from 0.016 to 79.2 µg/L [11,16,17].

Based on this literature data, it is evident that even under identical leaching conditions, cups from different manufacturers exhibit diverse leaching behaviors. These findings suggest that the observed variability in leachate composition could be attributed to differences in material quality, manufacturing processes, or experimental conditions across studies.

### 3.4. Health Risk Assessment

#### 3.4.1. Average Daily Intake

Based on the exposure parameters outlined in Table 1 and the elemental composition of leachates from paper and plastic cups (Table 2), the average daily intake (*ADI*) of each investigated element was estimated for adults and children (Equation (1)). The calculated ADI values, presented in Table 4, provide a comparative analysis of exposure levels across age groups and cup materials. Among the determined elements, As, Cd, Cr, and Pb are classified as carcinogens according to the categorization of the International Agency for Research on Cancer (IARC) [26]. The ADI values of these elements, as shown in Table 4, were calculated assuming an average human life expectancy (AT) of 70 years, equivalent to 70 × 365 days. It can be noted that the values of ADI differ between adults and children, as well as between paper and plastic cups. Among the determined elements, Ba and Pb demonstrate particularly notable differences in ADI values between paper and plastic cups. For adults, the ADI of Ba is considerably higher in plastic cups (0.149 µg/kg_bw_/day) compared to paper cups (0.014 µg/kg_bw_/day), reflecting more than a tenfold increase. Similarly, the ADI of Pb in plastic cups is substantially greater, reaching 0.206 µg/kg_bw_/day for adults, in contrast to 0.008 µg/kg_bw_/day in paper cups. These disparities underline the increased leaching of Ba and Pb from plastic cups, raising concerns about their suitability for hot beverage consumption. Across all elements, children show higher relative ADI values than adults due to their lower body weights. This amplifies the potential risk for children when exposed to even low levels of these elements. Moreover, plastic cups generally exhibit higher ADI values for most elements compared to paper cups, indicating greater leaching of contaminants from plastic.

#### 3.4.2. Hazard Quotient and Excess Lifetime Cancer Risk

The health risk metrics hazard quotient (HQ) and excess lifetime cancer risk (ELCR) were applied to assess the risk associated with exposure to leachates from disposable paper and plastic cups. These metrics provide insight into both non-carcinogenic and carcinogenic risks associated with long-term exposure. The calculated HQ values for both adults and children age groups, as presented in Table 5, remain consistently below unity for the investigated elements except for Pb. This indicates that, under the studied experimental conditions, the exposure levels of the examined elements, except Pb, leached from the tested paper and plastic cups are unlikely to pose non-carcinogenic health effects. On the other hand, Pb exhibited notably elevated HQ values in all types of cups, being much higher in plastic cups compared to paper cups. Its HQ values for adults and children in paper cups recorded 0.638 and 0.330, respectively, while they reached 17.2 for adults and 8.89 for children in plastic cups. These findings underscore the marked non-carcinogenic risk of Pb exposure associated with paper and plastic cups, which may be attributed to material composition or manufacturing processes. The elevated HQ values of Pb may be attributed to its introduction in plastic materials as an efficient heat stabilizer or pigment, or its introduction as a contaminant during the production process [27].

The ELCR was estimated for elements with available SFO values. Among the investigated elements, only four, namely As, Cr, Cd, and Pb, have SFO values reported in the literature. The calculated ELCR values for these four elements were calculated for adults and children, and the data are listed in Table 5. It can be noted that the ELCR values of all elements are between 1.74 × 10^−8^ and 1.76 × 10^−6^. While most values remain below the U.S. EPA’s target risk level of 1.0 × 10^−6^, the maximum ELCR of 1.76 × 10^−6^ marginally exceeds this benchmark. Nevertheless, the overall carcinogenic risk associated with long-term exposure to these elements remains low for both adults and children.

#### 3.4.3. Limitation of the Current Study

The current study has several limitations that should be addressed in future investigations. It is important to figure out if the leaching conditions employed in the current experiments are simulations designed to mimic the use of disposable cups with hot water. However, real-world conditions may involve a variety of beverages and foods with differing compositions, temperatures, and pH levels, which could influence the degree of potentially toxic elements leachability. Future studies should consider investigating these variables to provide a more comprehensive assessment of exposure risks under diverse real-life scenarios. Another limitation is the limited range of disposable cup types that was explored. Moreover, expanding the scope of work to include more diverse disposable product types could broaden insights. Additionally, this study focused on chemical elements, leaving other potential contaminants, such as organic substances and microplastics, uncovered. In this study, we did not characterize the polymeric composition or specify the manufacturer’s recommended temperature range for paper cup lining material. Since many lining materials are engineered for use only up to a specific temperature, exposure at higher temperatures—or prolonged contact with hot beverages—could accelerate polymer degradation and increase the leaching of toxic substances. Future work will involve detailed polymer identification and systematic migration testing across a range of time–temperature conditions to quantify how deviations from design specifications affect leaching potential. Furthermore, future studies should assess the fate of single-use products under diverse environmental conditions as well as their impact on various ecosystems. Addressing these gaps would enhance the robustness and applicability of findings, contributing to a comprehensive understanding of the health risks and environmental impacts associated with disposable food containers.

## 4. Conclusions

This study demonstrates that disposable paper and plastic cups, when exposed to hot water, release measurable quantities of potentially toxic elements into leachates, raising concerns regarding environmental and public health risks. Twelve elements, namely As, Ba, Cd, Co, Cr, Cu, Mn, Mo, Pb, Sb, V, and Zn, were examined. The substantial variability among cups of the same type suggests that raw material quality and manufacturing processes significantly influence leaching behavior.

The excess lifetime cancer risk (ELCR) analysis demonstrated that the carcinogenic risk from elements such as As, Cd, Cr, and Pb remains within the safe range. Although the hazard quotient (HQ) for most elements remained below unity, indicating negligible non-carcinogenic risks under the study conditions, Pb posed a marked non-carcinogenic risk, especially in plastic cups, with HQ values exceeding acceptable limits.

The variability in mass fractions of potentially toxic elements leached from disposable cups can be attributed to differences in raw material quality and manufacturing processes. These aspects emphasize the need for standardized manufacturing practices to reduce leaching variability and enhance consumer safety.

Future investigations should be conducted to examine the leachability of potentially toxic elements under real-life conditions of use, including varying temperatures, exposure times, and beverage types, to provide a comprehensive understanding of the factors influencing contaminant release.

Overall, the findings of the current studies bring to light that disposable cups should be treated as an emerging source of food chemical pollution and urge the need for deeper investigations to assess their impact on the environment, ecosystems, and human health. Moreover, the findings underscore the need for stricter regulatory frameworks to monitor and control the materials used in the production of disposable cups, particularly plastic ones. Manufacturers should prioritize improving production quality to reduce the leaching of harmful elements, while consumers are advised to minimize the use of disposable plastic products for hot beverages. These results emphasize the importance of transitioning towards safer, more sustainable materials for disposable food containers.

## Figures and Tables

**Table 1:** Exposure parameters for different age groups.

Age	IR, L/Day	EF, Days/Year	ED, Years	BW, kg	AT, Days	
					Carcinogen	Non-Carcinogen
Children (up to 6 years)	0.50	365	6	15	25,550	2180
Adults (>6 years)	1.13	365	24	70	25,550	8760

**Table 2:** Elemental composition of leachates from paper and plastic cups exposed to hot water (initial temperature ~ 95–100 °C) for 15 min.

Element	Mass Fraction, µg/L							
	Paper Cups (*n* = 20)				Plastic Cups (*n* = 5)			
	Median	STDEV	Min	Max	Median	STDEV	Min	Max
As	0.045	0.015	0.026	0.055	0.019	0.090	0.014	0.033
Ba	0.891	0.389	0.788	1.69	9.20	6.33	4.75	14.5
Cd	0.016	0.041	0.004	0.107	0.041	0.022	0.014	0.069
Co	0.009	0.004	0.004	0.011	0.014	0.002	0.007	0.071
Cr	0.610	0.312	0.021	0.704	0.555	0.43	0.131	1.11
Cu	0.325	0.443	0.126	1.30	0.951	0.629	0.110	3.31
Mn	1.592	0.310	1.28	2.08	3.372	0.134	0.061	4.60
Mo	0.055	0.042	0.025	0.085				
Pb	1.38	9.94	0.583	22.9	37.2	19.0	20.1	42.3
Sb	0.009	0.003	0.004	0.010				
V	0.017	0.017	0.010	0.054	0.053	0.033	0.011	0.092
Zn	14.4	12.8	9.21	45.0	10.8	9.50	9.23	31.5

**Table 3:** Literature data on leaching of potentially toxic elements from paper and plastic cups.

Element	Mass Fraction, µg/L *				
	Paper [13]	Paper [17]	Plastic [17]	Paper [11]	Plastic [16]
As	N.A.	N.A.	N.A.	<0.01–0.10	N.A.
Ba	N.A.	0.462 ± 0.054	0.436 ± 0.029	N.A.	N.A.
Cd	0.98–18.8	0.005 ± 0.002	0.003 ± 0.001	N.A.	3.00
Co	N.A.	0.022 ± 0.014	0.015 ± 0.006	<0.001–0.51	N.A.
Cr	ND–32.1	0.033 ± 0.019	0.029 ± 0.018	0.89–1.18	0.003
Cu	0.81–235	0.067 ± 0.035	0.076 ± 0.056	<0.01–1.80	0.002
Mn	3.66–514	0.367 ± 0.216	0.126 ± 0.033	N.A.	N.A.
Mo	N.A.	N.A.	N.A.	N.A.	N.A.
Pb	1.37–79.2	0.017 ± 0.001	0.016 ± 0.002	N.A.	15.5
Sb	N.A.	0.056 ± 0.004	0.055 ± 0.002	N.A.	N.A.
V	N.A.	0.015 ± 0.003	0.011 ± 0.002	1.06–1.55	N.A.
Zn	10.9–454	0.996 ± 0.023	1.16 ± 0.21	N.A.	N.A.

* N.A. refers to not available.

**Table 4:** Average daily intake of the determined elements for adults and children consuming hot beverages in paper and plastic cups.

Element	ADI, µg/kg_bw_/Day			
	Paper Cups		Plastic Cups	
	Adults	Children	Adults	Children
As	2.49 × 10^−4^	1.29 × 10^−4^	1.05 × 10^−4^	5.43 × 10^−5^
Ba	1.43 × 10^−2^	2.98 × 10^−2^	1.49 × 10^−1^	3.08 × 10^−1^
Cd	8.86 × 10^−5^	4.57 × 10^−5^	2.27 × 10^−4^	1.17 × 10^−4^
Co	1.44 × 10^−4^	2.98 × 10^−4^	2.26 × 10^−4^	4.69 × 10^−4^
Cr	3.37 × 10^−3^	1.74 × 10^−3^	3.07 × 10^−3^	1.59 × 10^−3^
Cu	5.25 × 10^−3^	1.09 × 10^−2^	1.53 × 10^−2^	3.18 × 10^−2^
Mn	2.57 × 10^−2^	5.33 × 10^−2^	5.44 × 10^−2^	1.13 × 10^−1^
Mo	8.88 × 10^−4^	1.84 × 10^−3^		
Pb	7.66 × 10^−3^	2.95 × 10^−3^	2.07 × 10^−1^	1.07 × 10^−1^
Sb	1.37 × 10^−4^	2.70 × 10^−4^		
V	2.66 × 10^−4^	5.53 × 10^−4^	8.56 × 10^−4^	1.77 × 10^−3^
Zn	2.33 × 10^−1^	4.84 × 10^−1^	1.75 × 10^−1^	3.64 × 10^−1^

**Table 5:** Hazard quotient (HQ) and excess lifetime cancer risk (ELCR) of potentially toxic elements in leachate of paper and plastic cups.

Element	Paper Cups				Plastic Cups			
	Adults		Children		Adults		Children	
	HQ	ELCR	HQ	ELCR	HQ	ELCR	HQ	ELCR
As	5.79 × 10^−5^	3.74 × 10^−7^	2.99 × 10^−5^	1.93 × 10^−7^	2.45 × 10^−5^	1.58 × 10^−7^	1.26 × 10^−5^	8.14 × 10^−8^
Ba	7.19 × 10^−5^		1.49 × 10^−4^		7.43 × 10^−4^		1.54 × 10^−3^	
Cd	8.86 × 10^−4^	3.37 × 10^−8^	4.57 × 10^−4^	1.74 × 10^−8^	2.27 × 10^−3^	8.62 × 10^−8^	1.17 × 10^−3^	4.45 × 10^−8^
Co	4.79 × 10^−4^		9.93 × 10^−4^		7.53 × 10^−4^		1.56 × 10^−3^	
Cr	1.13 × 10^−3^	1.69 × 10^−6^	5.81 × 10^−4^	8.71 × 10^−7^	1.02 × 10^−3^	1.54 × 10^−6^	5.29 × 10^−4^	7.93 × 10^−7^
Cu	1.31 × 10^−4^		2.72 × 10^−4^		3.84 × 10^−4^		7.96 × 10^−4^	
Mn	1.84 × 10^−4^		3.81 × 10^−4^		3.89 × 10^−4^		8.07 × 10^−4^	
Mo	1.78 × 10^−4^		3.68 × 10^−4^					
Pb	6.38×10^−1^	6.51 × 10^−8^	3.30 × 10^−1^	3.36 × 10^−8^	1.72 × 10^1^	1.76 × 10^−06^	8.89 × 10^0^	9.07 × 10^−7^
Sb	3.43 × 10^−4^		7.12 × 10^−4^					
V	5.28 × 10^−5^		1.10 × 10^−4^		1.70 × 10^−4^		3.52 × 10^−4^	
Zn	7.78 × 10^−4^		1.16 × 10^−3^		5.84 × 10^−4^		1.21 × 10^−3^	

## Data Availability

The data presented in this study are available on request from the corresponding author.

## Supplementary Materials

The following supporting information can be downloaded at: https://www.mdpi.com/article/10.3390/toxics13080626/s1, Figure S1: Sample of the investigated paper and plastic cups; Figure S2: Plastic thin film lining paper cups. Table S1: Minimum detection limits for the elemental analysis (LOD).
