# Supplementary material for: Leaching of Potentially Toxic Elements from Paper and Plastic Cups in Hot Water and Their Health Risk Assessment
Source: Toxics. 2025 Jul 26;13(8):626. doi: 10.3390/toxics13080626 (PMC12390227; doi:10.3390/toxics13080626)
Supplement: Supplementary file 1 [file toxics-13-00626-s001.zip › toxics-3746518-supplementary.pdf]

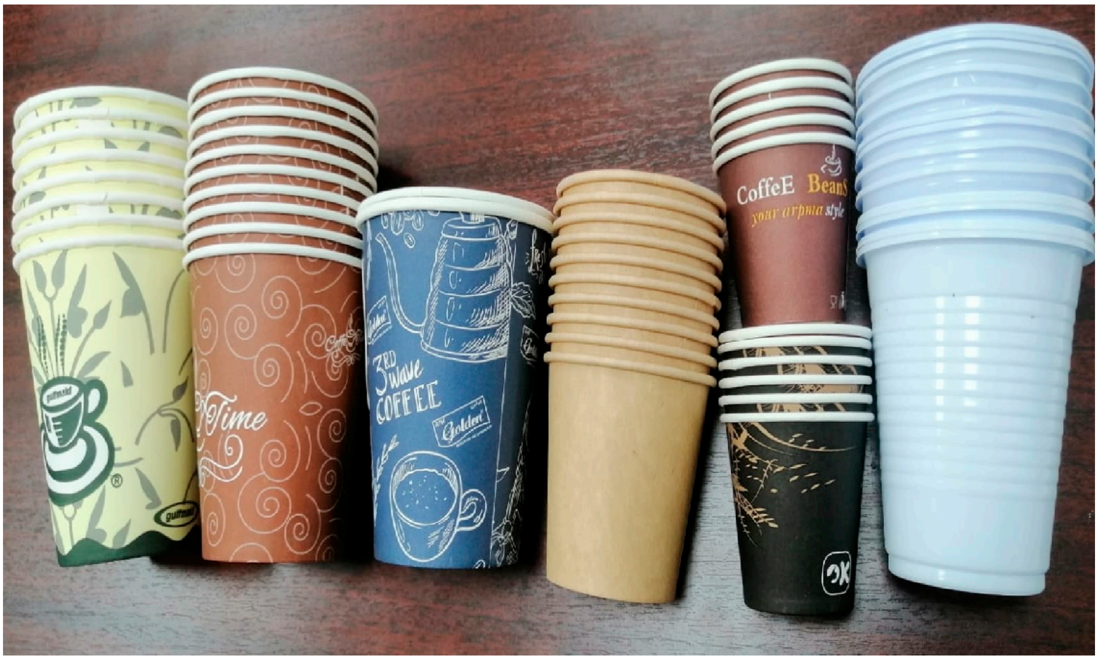

Figure S1. Sample of the investigated paper and plastic cups.

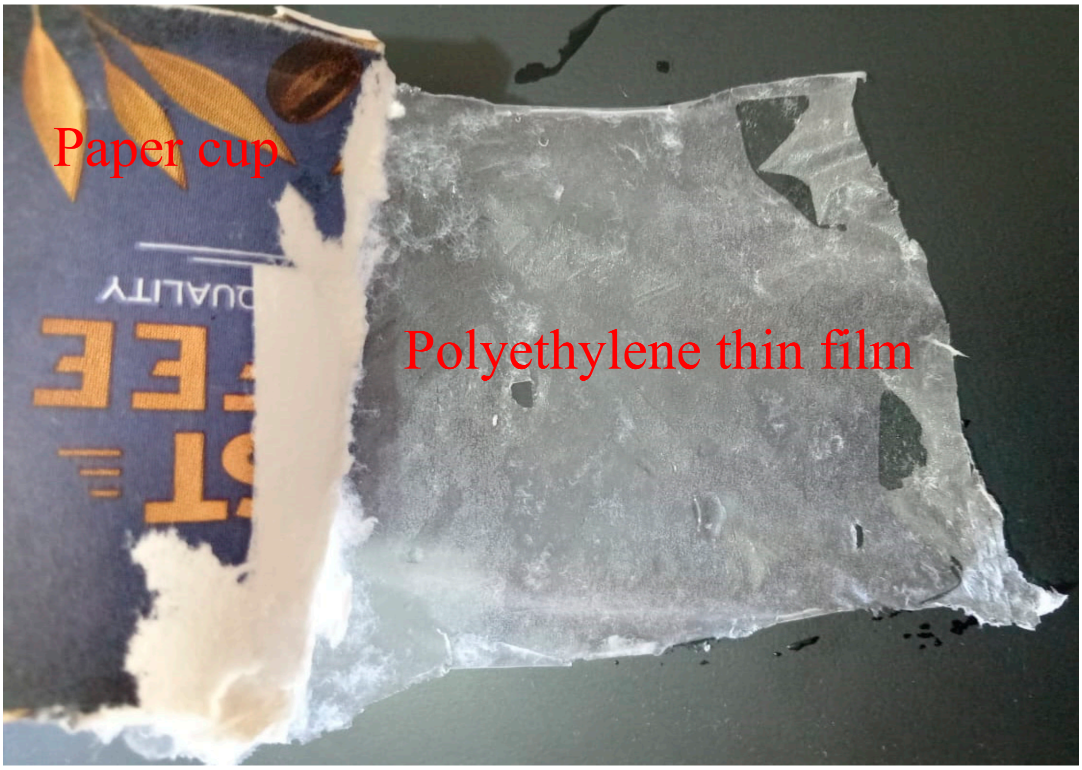

Figure S2. Plastic thin film lining paper cups.

Table S1. Minimum detection limits for the elemental analysis (LOD).

**Element**	**LOD (ppb)**
Sb	0.002
As	0.003
Ba	0.031
Cd	0.002
Cr	0.015
Co	0.001
Cu	0.005
Pb	0.032
Mn	0.047
Mo	0.014
V	0.007
Zn	0.154
